# The ACCELERATE Plus (assessment and communication excellence for safe patient outcomes) Trial Protocol: a stepped-wedge cluster randomised trial, cost-benefit analysis, and process evaluation

**DOI:** 10.1186/s12912-023-01439-x

**Published:** 2023-08-21

**Authors:** Mark Liu, Susan Whittam, Anna Thornton, Liza Goncharov, Diana Slade, Benjamin McElduff, Patrick Kelly, Chi Kin Law, Sarah Walsh, Vivien Pollnow, Jayde Cuffe, Jake McMahon, Christina Aggar, Jacqueline Bilo, Karen Bowen, Josephine S. F. Chow, Katharine Duffy, Bronwyn Everett, Caleb Ferguson, Steven A. Frost, Narelle Gleeson, Kate Hackett, Ivanka Komusanac, Sonia Marshall, Sharon May, Gemma McErlean, Gregory Melbourne, Jade Murphy, Joanne Newbury, Deb Newman, John Rihari-Thomas, Hayley Sciuriaga, Lauren Sturgess, Joanne Taylor, Karen Tuqiri, Elizabeth McInnes, Sandy Middleton, on behalf of the ACCELERATE Plus Project Team, Caitlin Alsop, Ainslie Cahill, Geoffrey Delaney, James Mackie, Kathryn Riddell, Mary Ryan, Christopher White, Rae Rafferty, Travis Brown, Joanne Chappelow, Sharon Curtis, Sarah Faulds, Jessica George, Sheena Lagat, Leanne Lee, Rebecca McEwin, Laura Richmond, Yvonne Steadward, Tara Doyle, Carmel Parker, Patty Zenonos

**Affiliations:** 1 Nursing Research Institute, St Vincent’s Health Network Sydney, St Vincent’s Hospital Melbourne, Australian Catholic University, De Lacy Building, 390 Victoria Street, Darlinghurst, NSW 2010 Australia; 2 School of Nursing, Midwifery and Paramedicine, Australian Catholic University, 40 Edward Street, North Sydney, NSW 2060 Australia; 3 St Vincent’s Health Network Sydney, 390 Victoria Street, Darlinghurst, NSW 2010 Australia; 4 Institute for Communication in Healthcare, Australian National University, Baldessin Precinct Building, 110 Ellery Crescent, Acton, ACT 2601 Australia; 5 School of Public Health, University of Sydney, Edward Ford Building, A27 Fisher Road, Camperdown, NSW 2006 Australia; 6 National Health and Medical Research Council Clinical Trials Centre, University of Sydney, Medical Foundation Building, 92-94 Parramatta Road, Camperdown, NSW 2050 Australia; 7 St Vincent’s Hospital Melbourne, 41 Victoria Parade, Fitzroy, VIC 3065 Australia; 8 Southern Cross University, Military Road, East Lismore, NSW 2480 Australia; 9 Northern NSW Local Health District, Crawford House, Hunter Street, Lismore, NSW 2480 Australia; 10 South Western Sydney Local Health District, Liverpool Hospital Eastern Campus, Corner of Lachlan and Hart Streets, Liverpool, NSW 2170 Australia; 11 Ingham Institute for Applied Medical Research, 1 Campbell Street, Liverpool, NSW 2170 Australia; 12 University of Wollongong, Northfields Avenue, Wollongong, NSW 2522 Australia; 13 Lismore Base Hospital, 60 Uralba Street, Lismore, NSW 2480 Australia; 14 South Eastern Sydney Local Health District, The Sutherland Hospital and Community Health Service, Corner The Kingsway and Kareena Road, Caringbah, NSW 2229 Australia; 15 Sydney Local Health District, King George V Building, Missenden Road, Camperdown, NSW 2050 Australia; 16 Fairfield Hospital, Polding Street and Prairie Vale Road, Prairiewood, NSW 2176 Australia; 17 The Sutherland Hospital, Corner The Kingsway and Kareena Road, Caringbah, NSW 2229 Australia; 18 Royal Prince Alfred Hospital, 50 Missenden Road, Camperdown, NSW 2050 Australia; 19 St George Hospital, Gray Street, Kogarah, NSW 2217 Australia; 20 Prince of Wales Hospital, 320-346 Barker Street, Randwick, NSW 2031 Australia

**Keywords:** Evidence-based nursing, Implementation science, Patient safety, Nursing assessment, Clinical handover, Multidisciplinary handover communication, Randomised controlled trial, Cost–benefit analysis, Process evaluation, Learning health system.

## Abstract

**Background:**

Nurses play an essential role in patient safety. Inadequate nursing physical assessment and communication in handover practices are associated with increased patient deterioration, falls and pressure injuries. Despite internationally implemented rapid response systems, falls and pressure injury reduction strategies, and recommendations to conduct clinical handovers at patients’ bedside, adverse events persist. This trial aims to evaluate the effectiveness, implementation, and cost–benefit of an externally facilitated, nurse-led intervention delivered at the ward level for core physical assessment, structured patient-centred bedside handover and improved multidisciplinary communication. We hypothesise the trial will reduce medical emergency team calls, unplanned intensive care unit admissions, falls and pressure injuries.

**Methods:**

A stepped-wedge cluster randomised trial will be conducted over 52 weeks. The intervention consists of a nursing core physical assessment, structured patient-centred bedside handover and improved multidisciplinary communication and will be implemented in 24 wards across eight hospitals. The intervention will use theoretically informed implementation strategies for changing clinician behaviour, consisting of: nursing executive site engagement; a train-the-trainer model for cascading facilitation; embedded site leads; nursing unit manager leadership training; nursing and medical ward-level clinical champions; ward nurses’ education workshops; intervention tailoring; and reminders. The primary outcome will be a composite measure of medical emergency team calls (rapid response calls and ‘Code Blue’ calls), unplanned intensive care unit admissions, in-hospital falls and hospital-acquired pressure injuries; these measures individually will also form secondary outcomes. Other secondary outcomes are: i) patient-reported experience measures of receiving safe and patient-centred care, ii) nurses’ perceptions of barriers to physical assessment, readiness to change, and staff engagement, and iii) nurses’ and medical officers’ perceptions of safety culture and interprofessional collaboration. Primary outcome data will be collected for the trial duration, and secondary outcome surveys will be collected prior to each step and at trial conclusion. A cost–benefit analysis and post-trial process evaluation will also be undertaken.

**Discussion:**

If effective, this intervention has the potential to improve nursing care, reduce patient harm and improve patient outcomes. The evidence-based implementation strategy has been designed to be embedded within existing hospital workforces; if cost-effective, it will be readily translatable to other hospitals nationally.

**Trial registration:**

Australian New Zealand Clinical Trials Registry ID: ACTRN12622000155796. Date registered: 31/01/2022.

**Supplementary Information:**

The online version contains supplementary material available at 10.1186/s12912-023-01439-x.

## Background

Improving patient safety in hospital settings is an ongoing challenge [1]. Among hospitals in developed countries, roughly one in 20 patients are exposed to a preventable incident of harm [2]. The majority of patient deterioration incidents are, at least in part, due to infrequent or delayed collection of vital signs and subsequent rapid response system activation [3, 4]. Inadequate safety practices also contribute to falls and pressure injuries, which are two hospital-acquired complications that largely occur due to delayed or omitted risk identification and prevention strategies [5, 6]. Another major contributor to preventable patient harm is poor communication within and between clinical teams, with one of the most common and important risk points being clinical handovers [7]. In response, three longstanding global imperatives for improving patient safety are: the early detection of deterioration [8], the reduction of preventable hospital-acquired complications [9], and improving communication during clinical handover [10]. The Australian National Safety and Quality Health Service Standards reflect the importance of implementing systems for addressing: (i) effective early recognition and response to deterioration; (ii) integrated assessment and risk identification processes; and (iii) effective communication for safety between multidisciplinary teams (defined in this article as nursing and medical), patients, carers and families [11]. Such standards are also paralleled and reinforced worldwide [12].

Patient harm is a significant burden for healthcare systems internationally [1]. For Australia, the average treatment cost for each case of clinical deterioration is $AUD26,778 and extends length of stay by eight days [13], the average cost of each case of fall-related injury is $AUD6,669 and extends length of stay by four days [14], and the average cost of each case of pressure injury costs between $AUD11,409—24,771 and extends hospital length of stay by five to nine days (depending on injury stage, 2 to 4) [15]. In total, hospital acquired complications, inclusive of falls and pressure injuries, cost Australian hospitals an estimated $AUD4.1 billion, or 8.9% of total hospital expenditure [16]. Beyond the financial burden, the prevalence of preventable adverse events also increases patient and caregiver distress and negatively impacts perceptions of the trustworthiness of the healthcare system [17].

Nurses play a central role in patient safety by conducting physical assessments and communicating findings to the multidisciplinary team, and implementing and evaluating the effect of nursing clinical interventions. Minimal vital signs datasets inform physiological tracking systems that determine the need for urgent patient interventions [4], and aberrations in vital signs must be recognised and acted upon to trigger medical rescue models such as rapid response systems and medical emergency teams [18]. Such approaches have been shown to decrease in-hospital mortality; however, patients who meet rapid response criteria still often require admission to intensive care and typically exhibit advanced physiological deterioration [19]. Reliance on vital signs alone creates a critical gap in nursing practice, in that patient deterioration is approached reactively rather than proactively [20, 21] and nurses’ capacity for critical decision making may be reduced [22]. Shifting to comprehensive and systematic physical assessments has the potential to identify, address and mitigate clinical deterioration, falls, and pressure injuries earlier in the deterioration process [23–25].

Complementary to improving nursing assessment practices, effective nurse-to-nurse and nurse-to-medical officer communication is essential for patient safety [7]. Clinical handovers are one of the most frequent and significant communicative processes between clinicians [26], but an estimated 80% of adverse events involve miscommunication during handover [10]. Recognised as a priority internationally [27], the Australian National Safety and Quality Health Service Standards encourage structuring handover using clinical communication frameworks, and conducting handovers at the bedside to support patient and caregiver involvement [11]. Upon recognising potential risks to patient safety, effective multidisciplinary communication practices are an essential mechanism to facilitate early escalation and actioning of treatment pathways [28]. Contextual (e.g. ward/hospital culture, hierarchies within organisations/disciplines) and personal factors (e.g. individual attitudes, team dynamics) have been observed to influence whether or not concerns are raised, and must be overcome to achieve effective communication [29].

In response to these patient safety issues, the Assessment and Communication Excellence for Safe Patient Outcomes (ACCELERATE) feasibility study was conducted in 2021 consisting of a stepped-wedge cluster randomised trial and process evaluation [30]. The intervention – a nurse-led ward-level intervention consisting of a nursing comprehensive and systematic core physical assessment, structured patient-centred bedside handover, and improved multidisciplinary communication – was implemented in three hospitals across two Australian states. The intervention was found to be feasible both by the research team and participating clinicians. This was despite periods of COVID-19 restrictions that necessitated a shift to online remote facilitation during the study, testing the potential feasibility of this mode of delivery. Significant improvements were observed for nurses’ perceptions of ward safety, barriers to change, and workplace culture (manuscript in preparation). A process evaluation was conducted at trial conclusion that qualitatively explored implementation barriers and facilitators and indicated that intervention adherence could be improved with increased engagement with medical teams (manuscript in preparation).

Building upon the ACCELERATE feasibility study, the aim of the ACCELERATE Plus Trial is to evaluate effectiveness, implementation, and cost–benefit of an enhanced intervention for nursing core physical assessment, structured patient-centred bedside handover and improved multidisciplinary communication. The ACCELERATE Plus Trial will be delivered and facilitated remotely by the research team using a train-the-trainer model, emphasising integration with existing hospital structures, personnel, and resources.

## Methods

**Fig. 1:**
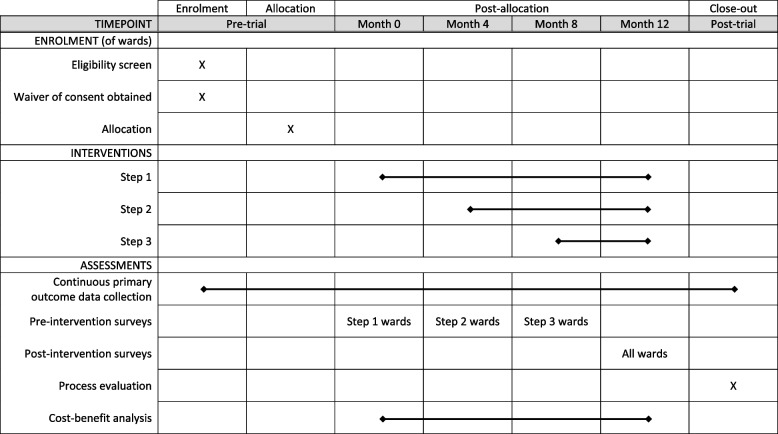
SPIRIT Diagram for schedule of enrolment, interventions, and assessments

The trial methods are reported according to the Standard Protocol Items: Recommendations for Interventional Trials (SPIRIT) [31], and the schedule items are shown in Fig. 1.

### Design

A pragmatic, hybrid type 1, stepped-wedge cluster randomised trial design will be conducted [32] with a cost–benefit analysis and qualitative process evaluation. The hybrid type 1 trial design allows the evaluation of intervention implementation concurrent to the primary clinical efficacy outcomes. Combining these data produces more useful information for decision-makers aiming to promote the more rapid translation of evidence to practice [33]. As our trial involves enhancement of current best practice, a stepped-wedge design is desirable as all participants eventually receive the intervention. The trial intervention, implementation and evaluation are informed by the Medical Research Council Framework for complex interventions [34] and evidence-based implementation strategies [35].

**Fig. 2:**
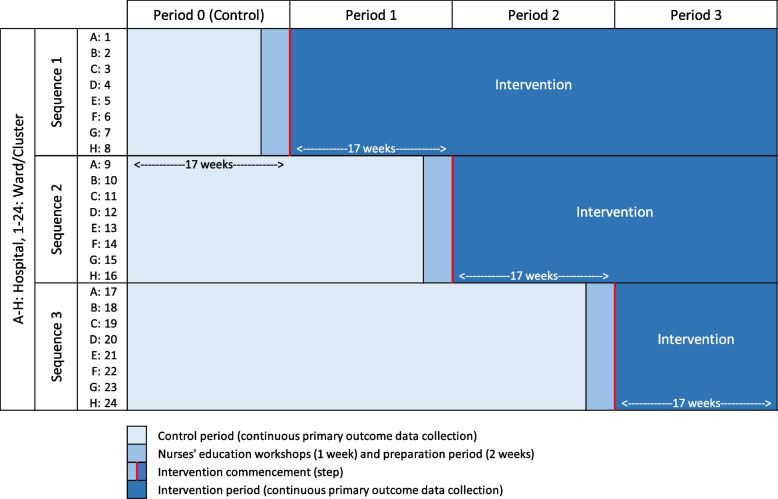
Stepped-wedge cluster randomised trial design

The trial intervention will be sequentially implemented over three periods of 17 weeks, totalling 51 weeks. A total of 24 wards will be enrolled to the trial, with eight sites each contributing three wards (Fig. 2).

### Setting

A convenience sample of eight hospitals will be selected. The majority of sites will be tertiary referral hospitals in metropolitan New South Wales.

### Ward eligibility criteria

General medical, surgical and rehabilitation wards with over 70% of registered and enrolled nurses on the ward as permanent staff, including permanent part-time and casual staff who work a minimum of four shifts per month, will be eligible for inclusion (reasonable staffing stability is important towards ward-level change [36]). Exclusion criteria are specialist units (e.g. intensive care, coronary care, emergency departments and mental health units), as well as outpatient clinics, operating theatres, diagnostic areas, or other non-general/acute care settings.

### Randomisation

Wards will be stratified by hospital and randomly allocated to one of the three sequences. Planned stratified allocation is shown in Fig. 2. Randomisation will be computer-generated and performed by a blinded researcher not involved in the trial.

## Clinical intervention

Intervention components are reported against the Template for Intervention Description and Replication (TIDieR) [37] in Supplementary Material 1. The intervention, refined from the ACCELERATE feasibility study [30], consists of three nurse-led components: core physical assessment, structured patient-centred bedside handover, and improved multidisciplinary communication.

### Core physical assessment

All registered and enrolled nurses will be required to conduct a comprehensive and systematic physical assessment for their allocated patients at the beginning of each shift. This consists of 16 core physical elements structured in the ‘A-E’ format (Table 1) [38, 39]. After this initial assessment, nurses will continue to perform vital sign monitoring per hospital policies and patient requirements.

**Table 1:** Core physical assessment elements, structured in A-E format [39]

A-E	Assessment Elements
Airway	1. Assess airway patency
Breathing	2. Measure respiratory rate 3. Evaluate work of breathing 4. Measure oxygen saturation
Circulation	5. Palpate pulse rate and rhythm 6. Measure blood pressure by auscultation 7. Assess urine output
Disability	8. Assess level of consciousness 9. Evaluate speech 10. Assess for pain
Exposure	11. Measure body temperature 12. Inspect skin integrity 13. Inspect and palpate skin for signs of pressure injury 14. Observe any wounds, dressings or drains, invasive lines 15. Observe ability to transfer and mobilise 16. Assess bowel movements

### Structured patient-centred bedside handover

Nurses will be required to perform nurse-to-nurse, shift-to-shift clinical handovers at the bedside at least once in every 24-h period and actively involve the patient and family/carer in the handover. Physical assessment findings will be communicated following the Introduction, Situation, Background, Assessment, Recommendations (ISBAR) protocol [40], and patient and family/carer interactions guided by the interactive protocol Connect, Ask, Respond, Empathise (CARE) protocol [41].

### Multidisciplinary communication

Bedside nurses will be encouraged to attend and actively participate in medical ward rounds and other multidisciplinary meetings by ‘speaking up’ on concerns identified from the core physical assessment, and collaboratively discuss plans of care and escalation requirements. ‘Speaking up’ has been defined as ‘assertive communication of patient safety concerns through information, questions or opinions where immediate action is needed to avoid patient harm’ [42].

## Implementation strategy

The intervention will be delivered using proven implementation strategies for changing clinician behaviour, consisting of: nursing executive site engagement; a train-the-trainer model for cascading facilitation [43, 44]; embedded site leads [45, 46]; nursing unit manager leadership training [47]; nursing and medical ward-level clinical champions [45, 48]; ward nurses’ education workshops [49]; intervention tailoring [50]; and reminders [51].

The ACCELERATE Plus Logic Model (Supplementary material 2) expands on these strategies and describes their determinants and mechanism of action [52]. Similar strategies have been successfully used in other nurse-led implementation research conducted by members of the research team [53–55].

### Nursing executive site engagement

Prior to and during the trial, the research team will engage with the directors of nursing (or equivalent/delegate) at the participating sites. Led by the trial clinical principal investigator (AT, Executive Director of Nursing at a New South Wales Specialty Health Network), this process involves advocacy for the key facilitative role of nursing executive support to drive the intervention locally.

### Train-the-trainer model for cascading facilitation using embedded site leads

**Fig. 3:**
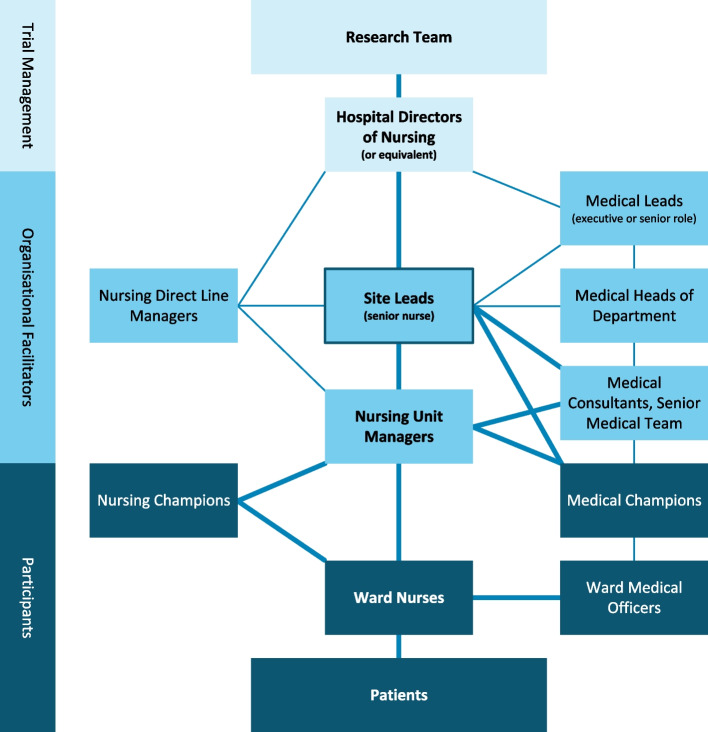
Cascading facilitation flowchart

Delivery and facilitation of the intervention will ‘cascade’ through stakeholders from all hospital managerial levels, from both nursing and medical disciplines, to ward clinicians and patients. The planned cascading facilitation is informed by the integrated Promoting Action on Research Implementation in Health Services Framework (i-PARIHS) [44, 56], and visually represented in Fig. 3.

Each participating hospital will nominate an internal nursing staff member to act as the site lead responsible for implementing the trial at their local site. Site leads will be nurses in an existing higher-grade position or senior clinical nurses who have high influence amongst the nursing discipline, with experience in nurse education and facilitating clinical practice change. Responsibilities will include: (i) engaging with nursing and medical management teams and presenting trial overviews, (ii) delivering nurses’ education workshops and additional top-up training as required, and (iii) supporting nursing unit managers to iteratively co-design their ward action plan with the ward clinicians to implement and embed the intervention.

A one and a half-day in-person train-the-trainer workshop with the site leads will be conducted two months prior to the start of the trial. Site leads will be briefed on the overall trial design, the intervention components, and their role within the trial, in particular, proposed engagement processes and training for delivering the nurses’ education workshops. The research team will remotely provide training, support, and resources for site leads to deliver the intervention and engage with site-specific stakeholders. Resources will include the workshop teaching material (PowerPoint presentation, demonstrative videos, and learning handouts), action plan templates, posters and lanyard cards to use as reminders on the ward, and intervention guides for optional internal audits.

Prior to and during the trial, all site leads will meet fortnightly with the research team via videoconference as a learning collaborative group to share progress, report risks, and discuss strategies for navigating barriers. Determined per site, regular internal meetings will be organised by the site lead to maintain engagement and support nursing unit managers (e.g., weekly to fortnightly action plan meetings with nursing unit managers and monthly progress reports with directors of nursing).

### Nursing unit manager leadership workshop

One month prior to each step, nursing unit managers of the participating wards in that step will participate in a one-day in-person workshop facilitated by an external consultant with extensive nursing leadership and education experience. The workshop aims to equip nursing unit managers with leadership and change management skills to lead and embed the intervention on their wards. A session of the workshop will be for nursing unit managers’ direct line managers to be informed of the trial and their role in providing operational oversight, addressing organisational barriers and serving as a liaison to other nursing and medical stakeholders.

### Nursing and medical ward-level clinical champions

Site leads will support nursing unit managers and their ward leadership team to identify and support clinical champions from both nursing and medical disciplines [57]. Champions will be individuals most likely to adopt the intervention early and act as role models to the ward, in particular to promote the multidisciplinary communication.

The previous ACCELERATE feasibility study involved medical heads of department during the initial engagement prior to each step and medical engagement at the ward team level. To strengthen interprofessional collaboration, the ACCELERATE Plus Trial will augment medical engagement by requesting each site to nominate a medical lead (medical executive or similar). This will assist in engaging with participating wards’ medical heads of department to identify medical champions and provide operational support. With the support of the site lead, nursing unit managers will collaborate with medical heads of department to facilitate the attendance of nurses in medical ward rounds.

### Ward nurses’ education workshops

A fortnight prior to the commencement of each step, site leads will conduct a two-hour workshop for nurses on the participating ward. The workshop will be repeated over five consecutive days to maximise attendance, with additional education sessions as required. These workshops will cover the three intervention components and facilitate discussion around anticipated ward-specific factors, specifically enablers, barriers, and proposed solutions.

### Intervention tailoring

The core physical assessment and medical engagement components of the trial may be tailored to the ward specialty and context. Wards with clinical specialties will be encouraged to tailor the core physical assessment by incorporating clinically specific elements in addition to the core 16 elements (e.g. cardiology wards may decide to incorporate arrhythmia assessment). To improve medical engagement, site leads and nursing unit managers will collaboratively determine any tailored approaches for facilitating nurse participation at medical ward rounds according to the multidisciplinary workflow of the ward.

### Reminders

The posters, lanyard cards and demonstrative videos provided by the research team can be used as reminders on the ward of the intervention components.

## Consumer involvement

Involvement of consumers is outlined in Table 2, according to the Guidance for Reporting Involvement of Patients and the Public 2 – Short Form (GRIPP2-SF). The trial will involve consumer representation from three separate organisations (a specialty health network, a local health district and a research translation centre).

**Table 2:** Consumer involvement reported according to GRIPP2-SF [58]

Section	Description
Aim:	To collaboratively involve consumers as research partners at all trial stages
Methods:	Recruitment occurred either during the previous feasibility study or at the initial stages of trial development. Consumers will be offered appropriate compensation [59]
Results:	Consumers will contribute by: - Ensuring trial aims, methods, intervention components and outcomes are aligned with patient and public interests - Providing a consumer perspective and input for the intervention and its implementation - Contributing to and reviewing trial documents, materials and resources - Providing a consumer perspective and advocating for patients at trial Management and Steering Committee meetings - Chairing Steering Committee meetings - Planning and presenting at the nursing unit manager leadership one-day workshop - Interpreting findings and developing publications - Advising on the future upscale of the intervention
Discussion:	Consumers have been embedded as feasible, with respect to: - Clinical and research knowledge - Consumers’ availability

## Outcome measures

## Primary

The primary outcome will be a composite measure of: (i) medical emergency team calls (consisting of: a) rapid response system calls, i.e. patients identified as potentially/actually clinically deteriorating, and b) ‘Code Blue’ calls, i.e. medical emergency and cardiopulmonary arrest [30]), (ii) unplanned intensive care unit admissions (patients unexpectedly requiring transfer from participating wards to the intensive care unit), (iii) in-hospital falls, and (iv) hospital-acquired pressure injuries.

## Secondary

Individual measures from the primary composite measure above will be secondary outcomes: medical emergency team calls (separated into rapid response system calls and ‘Code Blue’ calls), unplanned intensive care unit admissions, in-hospital falls (separated into all falls and falls occasioning harm) and hospital-acquired pressure injuries (separated by staging [60]).

### Patients’ experience measures

Patient reported perceptions of safety and overall hospital experience will be calculated using the following validated tools (i) Patient Measure of Safety Questionnaire [61] and (ii) the Friends and Family Test [62].

### Nurses’ experience measures

Registered and enrolled nurses’ perceptions of safety attitudes, organisational readiness to change, barriers to physical assessment, staff engagement and multidisciplinary collaboration will be quantified using the following validated tools: (i) Safety Attitudes Questionnaire Short Form (Teamwork Climate and Safety Climate subscales) [63], (ii) Organisational Readiness to Change Assessment (Context Assessment subscales) [64], (iii) Barriers to Nurses’ use of Physical Assessment Scale [65], (iv) Utrecht Work Engagement Scale-17 [66], and (v) Interprofessional Collaboration Scale [67].

### Medical officer’ experience measures

Medical officers’ perceptions of safety attitudes and multidisciplinary collaboration will be measured using the following validated tools (i) Safety Attitudes Questionnaire Short Form [63] and (ii) Interprofessional Collaboration Scale [67].

## Procedure

### Patients

All patients who are present on participating wards for 48 h or longer during the pre/post intervention survey periods will be invited to participate in the patient survey. Patients will be ineligible if they are: less than 18 years old, non-English speaking, or too unwell or cognitively impaired to participate (as determined by the primary nurse or site lead). Patients will be approached a maximum of three times to participate in the survey. If present, visiting family members and carers may assist patients to complete the survey.

### Nurses

All registered and enrolled nurses (including nursing unit managers) who have worked on participating wards for two weeks or more will be invited to complete the nurse survey, including part-time, temporary contract and casual staff who work 4 shifts or more per month. Agency staff employed by an external provider and assistants in nursing will not be eligible to participate.

### Medical officers

All medical staff (i.e. interns, residents, registrars and consultants) who have been based on participating wards for 2 weeks or longer will be invited to participate in the survey.

## Data collection

### Primary outcome data

Primary outcome data of medical emergency team calls (rapid response calls and ‘Code Blue’ calls), unplanned intensive care unit admissions, in-hospital falls, hospital-acquired pressure injuries, and patient demographic data (age, sex, comorbidity information, and time on ward) will be retrieved from routinely collected hospital data. Data will be collected for the trial period and the preceding three years (i.e. January 2020 – December 2023). Where routinely collected data does not clearly identify the location where a patient experienced an outcome event, medical record chart audits may be undertaken.

### Experience measures surveys (patient, nurse, medical officer)

Prior to each step and at the conclusion of the trial, patient, nurse, and medical officer experience measures will be obtained via paper-based self-administered surveys. Surveys will be distributed to intervention ward participants by site leads or ward-level clinical champions. For patients who have vision or physical impairments, but otherwise are eligible and willing to participate, site leads or caregivers may provide assistance in completing the survey. Return of completed surveys will constitute informed consent. Surveys will also contain some brief demographic questions: patient surveys will ask time on ward, age, sex, previous admission history and the 0–100 overall health visual analogue scale from the EuroQol 5-Dimensions Questionnaire [68]; nurse and medical officer surveys will ask their position title and how long they have worked on the participating ward.

## Data analysis

We expect to collect data from 48,960 patient episodes of care representing 244,800 bed days. With this sample, and with assumptions based on unpublished data for falls occasioning harm, pressure injuries and rapid response calls (intra-cluster correlation = 0.03–0.08, baseline rate = 2–8 incidents/1000 bed days) we will be powered to detect difference in the rate of incidents of 12–28% from pre- to post-intervention [30].

The data analysis period will extend across 68 weeks, comprising 51-week trial period and an additional 17-week control period prior to the first step (Fig. 2). All patients admitted or transferred to one of the study wards during the trial period will be included in the primary analysis. While outcome data are collected continuously, for analysis, time will be categorised per week. Days in weeks prior to the ward commencing the intervention will be counted as pre-intervention, with the exception of the three weeks immediately prior which will be omitted from the analysis as a period for implementation of the intervention (one week of nurses’ education workshops and two weeks of preparation). The three-year set of pre-trial primary outcome data will be used to investigate seasonal or temporal effects, or unusual variations in routinely collected data. Patient age, sex, comorbidity information and admission/transfer data will be used to adjust for variation in patient demographics between wards.

Patient outcome data will be modelled as counts using Poisson regression with an offset for time on ward. Using a Generalised Linear Mixed Model (GLMM) approach, we will use fixed effects for secular trend, and a random effect for ward. The primary outcome will be a composite measure of: (i) medical emergency team calls (inclusive of both rapid response system and ‘Code Blue’ calls); (ii) unplanned intensive care unit admissions; (iii) in-hospital falls; and (iv) hospital-acquired pressure injuries.

Patient, nurse and medical officer experience measures will be reported as means and standard deviations of overall component scores, and changes from pre- to post-intervention will be tested using a GLMM with a Poisson link function adjusting for respondent age, tenure (if appropriate) and correlation within ward. We will conduct secondary analyses: i) to investigate changes in outcomes over time, ii) perform the above with additional fixed effects for patient demographics (age, sex, comorbidity), and iii) model differences in primary outcome components by level of comorbidity. A statistical analysis plan pre-specifying all analyses will be formulated and finalized prior to the study data lock.

All analysis will be conducted in the R language for statistical computing, using the lme4 package for generalised linear mixed models [69].

### Cost–benefit analysis

Economic evaluation of the ACCELERATE Plus intervention compared with usual care will be assessed using a cost–benefit analysis. Resource use and cost of intervention will incorporate the involvement of staff, equipment hire and other materials for clinician training and related activities. The intervention’s effect on the primary outcome (medical emergency team calls, unplanned intensive care unit admissions, falls and pressure injuries) will be valued in monetary terms using the Australian unit costs obtained from Australia’s Independent Health and Aged Care Pricing Authority (formerly known as Independent Hospital Pricing Authority (IHPA)) and previous published/unpublished work [9]. Results for the cost–benefit analysis will be reported as the benefit-to-cost ratio; if the benefit-to-cost ratio is greater than 1, the intervention will be considered of value. To examine the robustness of results, sensitivity analyses will be conducted.

### Process evaluation

A process evaluation will be undertaken at the conclusion of the trial to understand the factors that influence intervention uptake and future sustainability by exploring the first-hand experiences of key stakeholders and participants. As outlined in Table 3, different participant groups will be invited to participate in either individual or group interviews. To maximise variation of trial contexts, we will purposively sample to include participants according to hospital/ward characteristics and prior involvement in the ACCELERATE feasibility study. Provisional sample size is guided by the scope of the research question, anticipated quality of data and pragmatics of recruitment [70].

**Table 3:** Planned process evaluation participants

Participant group	Target N	Interview type
Project team	2–5	Individual or group interviews
Site leads	8	Group interviews (2 groups of 4)
Directors of nursing^a^	8	Individual or group interviews
Nursing unit managers	15	Group interviews (5 groups of 3)
Ward nurses	20–40	Group interviews (5 groups of 4–8)
Medical officers^b^	Minimum of 5	Individual or group interviews

^a^Or equivalent/delegate

^b^Interns, residents, registrars and/or consultants

Data collection and analysis will be guided by the Normalisation Process Theory. Concepts from this theory cover individual, collective, and contextual factors that are associated with changing practices in complex healthcare settings, including: coherence (i.e. meaning and sense-making by participants), cognitive participation (i.e. commitment and engagement by participants), collective action (i.e. the work participants do to make the trial function) and reflexive monitoring (i.e. participants reflect on or appraise the trial) [71]. Members of the research team not involved in delivering the intervention will conduct the interviews. Participants will provide written informed consent to participate. Interview guides will consist of open-ended questions about participants’ role in the trial and intervention implementation, individual and contextual barriers and facilitators and perceptions on intervention sustainability, using the Normalisation Process Theory concepts as guiding prompts. Interviews will be digitally recorded, transcribed verbatim and thematically analysed [72]. Codes will firstly be identified openly, using the Normalisation Process Theory as a sensitising framework, followed by code reduction and the noting of converging data within or across sites and clinical disciplines. Subsequently, themes and subthemes will be generated.

## Discussion

The ACCELERATE Plus Trial aims to evaluate the effectiveness, implementation, and cost–benefit of an intervention for improving patient safety in acute care general ward settings consisting of nursing core physical assessment, structured patient-centred bedside handover and improved multidisciplinary communication. Current physical assessment practices predominantly consist of nurses completing minimal sets of vital signs which serve as criteria for activating medical emergency team calls [18]. Such models are reactive, often triggered late after the patient has already begun to deteriorate [20], and minimal vital signs sets are not designed to adequately assess the risk of falls and pressure injuries, nor does it encourage or facilitate the comprehensive assessment that is imperative for a summation of a patient’s clinical condition during handover [25]. Furthermore, current communication practices often do not actively include the patient or carers, which increases the likelihood of miscommunication and subsequent adverse events [10, 26]. We hypothesise that the effective implementation of a combined intervention that addresses these gaps will contribute to proactive safety practices that decrease medical emergency team calls, unplanned intensive care unit admissions, falls and pressure injuries.

Building on the findings from the ACCELERATE feasibility study [30], the current trial will seek to test the intervention across a new and larger sample of wards. Changing clinician behaviour is complex and multilayered, often requiring the involvement of many stakeholders ranging from directors to managers and ward clinical staff [73]. An important finding from the ACCELERATE feasibility study was the time and resource-intensive facilitation required by the researchers to implement the intervention. In addition, feedback from the ACCELERATE process evaluation suggests that locally embedded site leads are needed for optimal and accessible facilitation and ‘on-the-spot’ troubleshooting (manuscript in preparation). A recent study, which was similarly implemented and nurse-led, demonstrates the effectiveness of internal hospital staff facilitation [74]. External facilitation by training and supporting site leads embedded at each site is also a pragmatic approach for overcoming COVID-19 related restrictions for in-person access, as well as geographical barriers with interstate and regional sites. Testing this mode of delivery will provide valuable insight into the effectiveness of a less researcher-intensive method for translating the intervention to future sites. Having site leads with senior status and local influence delivering the intervention may also be beneficial for intervention sustainability.

Multidisciplinary collaboration between nurses and medical officers is an important factor for intervention uptake and adherence. The ACCELERATE feasibility study involved medical heads of department during the initial engagement prior to each step. For the current trial, engagement with medical teams will be enhanced so that all organisational levels (ward clinicians and management, departmental heads, and hospital directors) for both nursing and medical disciplines will be informed of the trial and potential areas for support.

### Strengths and limitations

The characteristics of the eight participating sites (hospital size, state, and geographical classification) increases the likelihood that, if effective, the findings will be generalisable and can be readily translated to other similar hospitals for upscale. Culturally and linguistically diverse patients however will likely be underrepresented, as this trial will not have adequate resourcing for interpretive services. A notable consideration is that intervention adherence will not be quantitatively measured due to the scale of the trial. Rather, the trial includes a process evaluation and cost–benefit analysis that will inform implementation factors and considerations for future translation. Another consideration will be the accuracy and consistency of routinely collected hospital data that comprise the primary outcome. However, using such data sources is also a strength as it provides a low-cost method of evaluating the effects of the trial, and can be accessed again at a later date to evaluate the long-term effects. The use of routinely collected hospital data, in combination with an externally facilitated implementation strategy and embedded site leads, aligns the ACCELERATE Plus Trial with the movement towards learning health systems for improving healthcare [75].

## Conclusion

If found to be effective, the ACCELERATE Plus Trial presents a model of care that will improve patient safety, nursing care, and both clinician and patient experience measures in a cost-effective manner. The addition of a trial process evaluation will allow the intervention and implementation strategy to be evaluated for readiness of translation to hospitals nationally. Findings from this trial may inform the design and evaluation of future trials that will aim to scale and/or sustain the intervention in different contexts.

### Supplementary Information

**Additional file 1:** **Supplemetary S1.** ACCELERATE Plus Trial Intervention, reported using the TIDieR Checklist.

**Additional file 2:** **Supplementary S2.** ACCELERATE Plus Trial Logic Model [52].

## Data Availability

Not applicable.
