# Correction: the ACCELERATE Plus (assessment and communication excellence for safe patient outcomes) Trial Protocol: a stepped-wedge cluster randomised trial, cost-benefit analysis, and process evaluation

**DOI:** 10.1186/s12912-023-01589-y

**Published:** 2023-11-24

**Authors:** Mark Liu, Susan Whittam, Anna Thornton, Liza Goncharov, Diana Slade, Benjamin McElduff, Patrick Kelly, Chi Kin Law, Sarah Walsh, Vivien Pollnow, Jayde Cuffe, Jake McMahon, Christina Aggar, Jacqueline Bilo, Karen Bowen, Josephine S. F. Chow, Katharine Duffy, Bronwyn Everett, Caleb Ferguson, Steven A. Frost, Narelle Gleeson, Kate Hackett, Ivanka Komusanac, Sonia Marshall, Sharon May, Gemma McErlean, Gregory Melbourne, Jade Murphy, Joanne Newbury, Deb Newman, John Rihari-Thomas, Hayley Sciuriaga, Lauren Sturgess, Joanne Taylor, Karen Tuqiri, Elizabeth McInnes, Sandy Middleton, on behalf of the ACCELERATE Plus Project Team

**Affiliations:** 1 Nursing Research Institute, St Vincent’s Health Network Sydney, St Vincent’s Hospital Melbourne, Australian Catholic University, De Lacy Building, 390 Victoria Street, Darlinghurst, NSW 2010 Australia; 2 School of Nursing, Midwifery and Paramedicine, Australian Catholic University, 40 Edward Street, North Sydney, NSW 2060 Australia; 3 St Vincent’s Health Network Sydney, 390 Victoria Street, Darlinghurst, NSW 2010 Australia; 4 Institute for Communication in Healthcare, Australian National University, Baldessin Precinct Building, 110 Ellery Crescent, Acton, ACT 2601 Australia; 5 School of Public Health, University of Sydney, Edward Ford Building, A27 Fisher Road, Camperdown, NSW 2006 Australia; 6 National Health and Medical Research Council Clinical Trials Centre, University of Sydney, Medical Foundation Building, 92‑94 Parramatta Road, Camperdown, NSW 2050 Australia; 7 St Vincent’s Hospital Melbourne, 41 Victoria Parade, Fitzroy, VIC 3065 Australia; 8 Southern Cross University, Military Road, East Lismore, NSW 2480 Australia; 9 Northern NSW Local Health District, Crawford House, Hunter Street, Lismore, NSW 2480 Australia; 10 South Western Sydney Local Health District, Liverpool Hospital Eastern Campus, Corner of Lachlan and Hart Streets, Liverpool, NSW 2170 Australia; 11 Ingham Institute for Applied Medical Research, 1 Campbell Street, Liverpool, NSW 2170 Australia; 12 University of Wollongong, Northfields Avenue, Wollongong, NSW 2522 Australia; 13 Lismore Base Hospital, 60 Uralba Street, Lismore, NSW 2480 Australia; 14 South Eastern Sydney Local Health District, The Sutherland Hospital and Community Health Service, Corner The Kingsway and Kareena Road, Caringbah, NSW 2229 Australia; 15 Sydney Local Health District, King George V Building, Missenden Road, Camperdown, NSW 2050 Australia; 16 Fairfield Hospital, Polding Street and Prairie Vale Road, Prairiewood, NSW 2176 Australia; 17 The Sutherland Hospital, Corner The Kingsway and Kareena Road, Caringbah, NSW 2229 Australia; 18 Royal Prince Alfred Hospital, 50 Missenden Road, Camperdown, NSW 2050 Australia; 19 St George Hospital, Gray Street, Kogarah, NSW 2217 Australia; 20 Prince of Wales Hospital, 320‑346 Barker Street, Randwick, NSW 2031 Australia

**Correction: BMC Nursing 22, 275 (2023)**

**https://doi.org/10.1186/s12912-023-01439-x**

Following publication of the original article [1], the authors reported errors in the authors and project team names of the ACCELERATE Plus Project Team.

The original article [1] has been corrected.
