# Supplementary material for: The ACCELERATE Plus (assessment and communication excellence for safe patient outcomes) Trial Protocol: a stepped-wedge cluster randomised trial, cost-benefit analysis, and process evaluation
Source: BMC Nurs. 2023 Aug 21;22:275. doi: 10.1186/s12912-023-01439-x (PMC10440862; doi:10.1186/s12912-023-01439-x)
Supplement: Supplementary file 1 — Additional file 1: Supplemetary S1. ACCELERATE Plus Trial Intervention, reported using the TIDieR Checklist. [file 12912_2023_1439_MOESM1_ESM.docx]

**Supplementary Material 1.** ACCELERATE Plus Trial Intervention, reported using the TIDieR Checklist

| **Item** | **Description** |
| --- | --- |
| 1. Brief name | The ACCELERATE Plus Trial |
| 2. Why | This trial aims to evaluate the effect of an organisational ward-level nursing patient safety intervention, measured using medical emergency team calls, unplanned intensive care unit admissions, in-hospital falls and hospital-acquired pressure injuries. The intervention consists of three components:   - Core physical assessment, - Structured patient-centred bedside handover, and - Improved multidisciplinary communication. |
| 3. What: materials | Education: didactic and interactive workshop using a PowerPoint presentation, demonstrative videos and learning handouts.  Ward-based materials: intervention posters, guides for optional internal audits and lanyard cards as reminders. |
| 4. What: procedures | **Core physical assessment**  All nurses will assess patients for the following elements for their allocated patients at the beginning of each shift:   - Airway: assess airway patency. - Breathing: measure respiratory rate; evaluate work of breathing; measure oxygen saturation. - Circulation: palpate pulse rate and rhythm; measure blood pressure by auscultation; assess urine output. - Disability: assess level of consciousness; evaluate speech; assess for pain. - Exposure: measure body temperature; inspect skin integrity; inspect and palpate skin for signs of pressure injury; observe any wounds, dressings or drains, invasive lines; observe ability to transfer and mobilise, assess bowel movements. - Wards may elect to incorporate additional elements specific to their clinical specialty (e.g. arrhythmia assessment for cardiology)   **Structured patient-centred bedside handover**  Nurse-to-nurse handovers will be conducted at the beside following protocols for:   - Physical assessment findings using the ISBAR protocol (introduction, situation, background, assessment, recommendations). - Interaction with patients, families and/or carers using the CARE protocol (connect, ask, respond, empathise).   **Multidisciplinary communication**  Nurses will be encouraged to attend and actively participate in medical ward rounds and other multidisciplinary meetings to raise concerns, and collaboratively discuss plans of care and escalation requirements. |
| 5. Who provided | The intervention will be delivered and facilitated using a train-the-trainer model for cascading facilitation:   - The **research team** (i.e. trial manager, research officers) will provide training and support to site leads. - **Site leads**, trained by the research team, will train ward nurses, facilitate medical engagement, and provide support at the hospital level. - **Nursing unit managers**, trained by an external consultant, will provide support at the ward level. - **Ward nurses**, trained by the site leads, will perform the intervention in collaboration with medical teams. - Hospital **managerial and executive staff** (from both nursing and medical disciplines) will provide operational support. |
| 6. How | **Site leads**: The research team will conduct a 1.5-day workshop on the overall trial, facilitation and intervention.  **Nursing unit managers**: An external consultant will conduct a 1-day workshop on leadership and change management skills.  **Ward nurses**: Site leads will conduct 2-hour workshops to train ward nurses in all intervention components. This is repeated over a 5-day period to maximise attendance.  **Intervention**: The intervention will be delivered by ward nurses according to action plans co-designed between nursing unit managers and site leads. |
| 7. Where | **Sites**: The study will take place at eight public hospitals across two Australian states, with varying characteristics and remoteness.  **Wards**: Participating wards (n=24, three from each hospital) will include general medical, surgical and rehabilitation. |
| 8. When and how much | Nurses will perform the core physical assessment **at the beginning of each shift**.  Nurses will perform a structured patient-centred bedside handover **at least once every 24 hours**.  Nurses will join **medical ward rounds and other multidisciplinary meetings** to raise concerns from the core physical assessment. |
| 9. Tailoring | Wards may incorporate additional elements to the core physical assessment according to the clinical specialty.  Wards will tailor mechanisms for engagement with local medical teams (e.g. formal/informal trial presentations and meetings). |
| 10. How well (planned) | Wards will be encouraged to internally monitor for adherence and report concerns to the research team or hospital management as appropriate. A post-trial process evaluation will qualitatively investigate factors that influence intervention adherence and uptake of implementation strategies. |
