# Supplementary material for: The ACCELERATE Plus (assessment and communication excellence for safe patient outcomes) Trial Protocol: a stepped-wedge cluster randomised trial, cost-benefit analysis, and process evaluation
Source: BMC Nurs. 2023 Aug 21;22:275. doi: 10.1186/s12912-023-01439-x (PMC10440862; doi:10.1186/s12912-023-01439-x)
Supplement: Supplementary file 2 — Additional file 2: Supplementary S2. ACCELERATE Plus Trial Logic Model [52]. [file 12912_2023_1439_MOESM2_ESM.docx]

**Supplementary Material 2.** ACCELERATE Plus Trial Logic Model (52)

**Nursing executive site engagement**

- Certain organisational barriers may only be addressed with executive support.
- Knowledge of executive support may motivate ward-level clinician behaviour change.

**Train-the-trainer model for cascading facilitation**

- Regular videoconference meetings with the research team allow site leads to collaboratively problem-solve barriers and share solutions.

**Nursing unit manager leadership training**

- Upskilling nursing unit managers with leadership and practice change skills increases their ability to support the intervention.
- Ward-level clinical champions will serve as role models.
- Co-designing action plans, with input from clinicians, site leads and nursing unit managers, may increase intervention adherence.

**Interprofessional collaboration**

- Actively involving both nursing and medical teams may facilitate and increase multidisciplinary communication.

**Ward nurses’ education workshops**

- Barrier and facilitator identification performed collectively with ward nurses may support intervention adherence.
- Tailoring the core physical assessment may increase the recognition of early signs of deterioration on specialty wards.

**Reminders**

- Posters and lanyards on wards may increase intervention fidelity.
- Workshop videos may be reused as reminders.

**Process**

- Early engagement will occur with influential stakeholders from each site.
- Implementation plans are developed in advance, in collaboration with consumers.

**Patient**

- Medical emergency team calls
- Unplanned intensive care unit admissions
- In-hospital falls
- Hospital-acquired pressure injuries
- Patient perceptions of safety and the Friends and Family Test (61, 62)

**Clinician**

Nursing and medical staff perceptions:

- Safety culture (63)
- Interprofessional collaboration (67)

Nurse perceptions:

- Organisational readiness to change (64)
- Barriers to physical assessment (65)
- Staff engagement (66)

**Clinical Intervention**

**Service**

The trial will:

- Promote patient-centred care.
- Involve all levels within the health service, from district nursing executives to ward nurses and medical officers.
- Promote multidisciplinary communication between teams.
- Facilitate nurses working to the top of their scope of practice.

**Outer setting**

- Trial encourages inter-organisational networks between participating hospitals and the local health districts.
- Research team has established links with external government patient safety bodies and state-based chief nurses.
- Intervention may require integration with existing policy.
- Patients are culturally and linguistically diverse.

**Inner setting**

- Sponsorship from directors of nursing and senior medical staff.
- Hospital/wards have unique contexts and cultures that may influence intervention uptake.
- Contexts may be unstable (i.e. changes to staffing, ward location, management and/or operations).

**Intervention characteristics**

- Based on best-practice research.
- Feasibility and acceptability previously demonstrated (30).

**Characteristics of individuals**

- Sites will nominate suitable i) internal staff as trial site leads, ii) ward clinical champions.
- Ward nurses possess requisite knowledge and skills to perform the intervention.
- Consumer representatives will aid in aligning the trial with patient and public interests at all trial stages.

1. **Core physical assessment** for all allocated patients at the start of each shift (39).
2. **Structured patient-centred bedside handover** using ISBAR (Introduction, Situation, Background, Assessment, Recommendations) and CARE (Connect, Ask, Respond, Empathise) (40, 41).
3. **Improved multidisciplinary communication** by attending and ‘speaking up’ at medical ward rounds (42).

**Nursing executive site engagement**

- Engagement will occur prior to trial commencement and for trial duration.

**Train-the-trainer model for cascading facilitation**

- Embedded site leads at each hospital (senior internal nursing staff member).
- 1.5-day face-to-face workshop to train site leads to deliver the intervention.
- Site lead learning collaborative group meeting with the research team.

**Nursing unit manager leadership training**

- 1-day face-to-face workshop for leadership skills and clinical practice change strategies.
- Identify nursing and medical ward-level clinical champions.

**Interprofessional collaboration**

- Clinician implementation team meetings between nursing and medical teams.
- Engagement processes tailored to ward context.

**Ward nurses’ education workshops**

- Interactive and didactic two-hour education workshop delivered by the site lead at each step.
- Barriers and facilitators identification tool.
- Workflow changes to facilitate intervention.
- Intervention can be tailored to ward specialty (for core physical assessment component).
- Additional ongoing education as required.

**Reminders**

- Intervention posters, lanyards and videos.

000

**Implementation Strategies**

**Implementation**

**Process evaluation** will explore factors that influence intervention uptake, using the Normalisation Process Theory (71).

**Cost-benefit analysis** will evaluate the economic value of the intervention compared to usual care.

**Outcomes**

**Mechanisms**

**Determinants**
